# Trends, Spatial Disparities, and Social Determinants of DTP3 Immunization Status in Indonesia 2004–2016

**DOI:** 10.3390/vaccines8030518

**Published:** 2020-09-10

**Authors:** Holipah Holipah, Asri Maharani, Sujarwoto Sujarwoto, Takuji Hinoura, Yoshiki Kuroda

**Affiliations:** 1 Faculty of Medicine, Universitas Brawijaya, Malang, Jawa Timur 65145, Indonesia; 2 Department of Public Health, Faculty of Medicine, University of Miyazaki, Miyazaki 889-1962, Japan; takuji_hinoura@med.miyazaki-u.ac.jp (T.H.); ykuroda@med.miyazaki-u.ac.jp (Y.K.); 3 Divisions of Neuroscience and Experimental Psychology, Faculty of Biology, Medicine and Health, The University of Manchester, Manchester M13 9PL, UK; asri.maharani@manchester.ac.uk; 4 Department of Public Administration, University of Brawijaya, Malang 65145, Indonesia; sujarwoto@ub.ac.id

**Keywords:** spatial disparities, social determinants, DTP3 immunization, multilevel analysis

## Abstract

Although 91% of 12–23-month-old children in Indonesia received at least one immunization in 2013, only 76% completed DTP3 immunization. This percentage is below the UNICEF and WHO recommended standards. Thus, this study aims to investigate trends, spatial disparities, and social determinants related to low coverage of DTP3 immunization in Indonesia. Using a multilevel approach, we analyzed data from 305,090 12–23-month-old children living across approximately 500 districts in Indonesia to study demand and supply factors determining DTP3 immunization status. We examined unique, nationally representative data from the National Socioeconomic Survey (*Survei Sosial Ekonomi Nasional* or *Susenas*) and Village Potential Census (*Potensi Desa* or *Podes*) from 2004 to 2016. The percentage of children receiving complete DTP3 immunization increased from 37.8% in 2004 to 75.9% in 2016. Primarily income, parity status, and education, showed influence on DTP3 coverage. Among individual-level factors, the presence of a professional birth attendant was the most influential factor. At the district level, the factors varied. Low progress in DTP3 immunization status in Indonesia is due to huge disparities across the country’s islands, in the density of health services, and in household socioeconomic status.

## 1. Introduction

Diphtheria was a severe, fatal infection before a vaccine was developed in 1940 and circulated worldwide [1]. The average worldwide incidence of diphtheria has declined dramatically from about 10,000 cases per year (2000–2004) to 5000 per year (2005–2009). However, the greatest numbers of individuals afflicted with diphtheria have been found in Indonesia, India, and Nepal [2]. Based on WHO data, there were 1192 diphtheria patients in Indonesia in 2012; this was the second highest number of cases in a single country after India, with 2525 patients in the same year [3]. In 2017, the incidence of diphtheria suddenly increased [4], leading the government of Indonesia to announce an outbreak of diphtheria; 613 cases of diphtheria across 95 regencies in 20 provinces were recorded between January and November of 2017. Eighty percent of the patients came from only seven provinces (East Java, West Java, Banten, Aceh, West Sumatra, DKI Jakarta, and Central Java) [5]. As many children in Indonesia were unvaccinated or partially vaccinated, that is, they received at least one dose of immunization but not full immunization on schedule, this outbreak of diphtheria was thought to be caused by a lack of immunization.

There are many islands in Indonesia, each island with its own culture and geographic characteristics. These different cultures and characteristics have an effect on differences in diphtheria, tetanus, and pertussis (DTP3) immunization status [6,7,8]. In 2001, the Indonesian government launched a decentralization of immunization facilities to improve immunization rates. This new policy committed to giving local governments the authority to regulate their own districts’ immunization policies as part of their health services. The government also publicized a strong commitment to improving immunization status. DTP3 for children was set as one of the routine basic immunizations in Indonesia. Children were therefore to receive a three-dose primary series of DTP3 at two, three, and four months; this was general protocol in Indonesia, in accordance with WHO recommendations [9]. In addition, new integrated health service posts (*posyandu*) and mobile health center (mobile *puskesmas*) programs, as well as a national immunization week, were intended to improve the proportion of immunization [6]. Recent reports state that the percentage of children aged 12–23 months who received complete DTP3 immunization was up to 76% in 2017 [10]. However, that proportion is below the WHO standard of 90%. Complete DTP3 immunization status was defined as the receipt of three doses of DTP3 immunization based on the national Expanded Program on Immunization (EPI) schedule.

We aimed to examine trends, spatial disparities, and social determinants related to the low rate of complete DTP3 immunization in Indonesia. In this study, we were able to access trends in DTP3 immunization in Indonesia during the 13 years between 2004 and 2016. We used the characteristics of the survey data, covering about 305,090 12–23-month-old children from approximately 500 districts over the 13-year period. The data consisted not only of individual socioeconomic factors but also district-level factors such as health service capacity. We focused on geographical characteristics, spatial disparities and social determinants, as well as on the role of district health services, to determine the factors that can improve children’s access to complete DTP3 immunization.

## 2. Material and Methods

### 2.1. Study Design and Data

A cross-sectional study was executed to determine the factors that promote higher rates of children immunized with the DTP3 vaccine. The main data were drawn from the National Socioeconomic Survey, *Survei Sosial Ekonomi Nasional* (*Susenas*) 2004–2016 and from the Village Potential Census, *Sensus Potensi Desa* (*Podes*) 2004–2016. *Susenas*, initiated in 1963, is an annual cross-sectional household survey administered by the Indonesian Statistical Bureau (*Badan Pusat Statistik*) and is a large, national, representative survey. Annually, the total number of individuals interviewed for *Susenas* is 1.25 million from 250,000 households in more than 500 districts across the country, and the data contain information on child immunization, individual education, employment, and household consumption expenditure. *Susenas* is therefore a main source of information in Indonesia, not only for the government but also for many international bodies including the World Bank [11,12].

*Podes* is a long-standing traditional system of data collection that contains information on infrastructures and services, including the numbers of hospitals, clinical service stations (health centers and health posts), medical doctors, and midwives [13]. The survey collects this data from every village in the country (*n* = 74,754). Information about each village’s facilities is typically derived from the village heads or *kepala desa/lurah* administration [13]. We used data from *Podes* 2004–2016.

### 2.2. Sociodemographic and District Factors

We used residence type, the presence of a professional birth attendant during labor, mother’s age at the time of survey, mother’s educational level, mother’s employment status, number of children, and household income as the sociodemographic factors. Residence types were separated into rural and urban areas. Regarding the presence of a professional birth attendant during labor, in one category, the mother was under the care of a professional birth attendant such as a midwife, nurse, or doctor, and in the other category, the mother was not supported by a professional birth attendant. Mother’s age was divided into three categories, ≤20 years, 21–30 years, and >30 years old. Mother’s educational level was categorized as no education/primary, secondary school, and higher than secondary school. Mother’s employment status was divided into those with paid employment and those without paid employment. Number of children was categorized as few (1–2 children), intermediate (3–5 children), or many (more than five children). Household income was measured by means of household expenditures over one year. Household expenditure is preferable to total income when the intention is to grasp long-term financial condition per capita, especially in developing countries such as Indonesia [14].

Local government health service capacity was measured by means of district factors supporting DTP3 immunization. Those factors were the number of health facilities (hospitals, health centers, and health posts) and health workers (medical doctors, nurses, and midwives) per 1000 population.

### 2.3. Statistical Analysis

The multilevel analyses were carried out in several steps. First, models were applied to the entire population. The regression model equation was as follows:

*E*_ij*_ = *ß*_o_ + Ʃ *ß_j_W_j_* + *ß_ij_X_ij_* + µ_*j*_ + ϵ_*ij*_

with a household *i* nested in district *j*, and where E_ij*_ = logit(*P*(E_ij*_ = 1) is a binary variable indicating a child with or without DTP3 immunization; *W_j_* is a set of district-level characteristics (i.e., hospitals per 1000 population, health centers (*puskesmas*) per 1000 population, health posts (*posyandu*) per 1000 population, medical doctors per 1000 population, and nurses and midwives per 1000 population); *X_ij_* is a set of household-level characteristics (i.e., mother’s socioeconomic characteristics, residential area, birth attendance, parity, and household income); *µ_j_* is a random intercept varying over districts with zero mean, and variance *σ_µ_*^2^; *ϵ_ij_* is normally distributed with zero mean and variance *σ_ϵ_*^2^.

To identify whether the effect of socioeconomic and health service factors varied according to place of residence, the analyses were stratified by residential island as well as rural and urban samples. Multilevel analyses were carried out using generalized linear latent and mixed models (GLLAMM) commands in Stata 14.0 software. Odds ratios were used to compare the magnitude of socioeconomic and health service determinants for DTP3 immunization status.

## 3. Results

**Figure 1:**
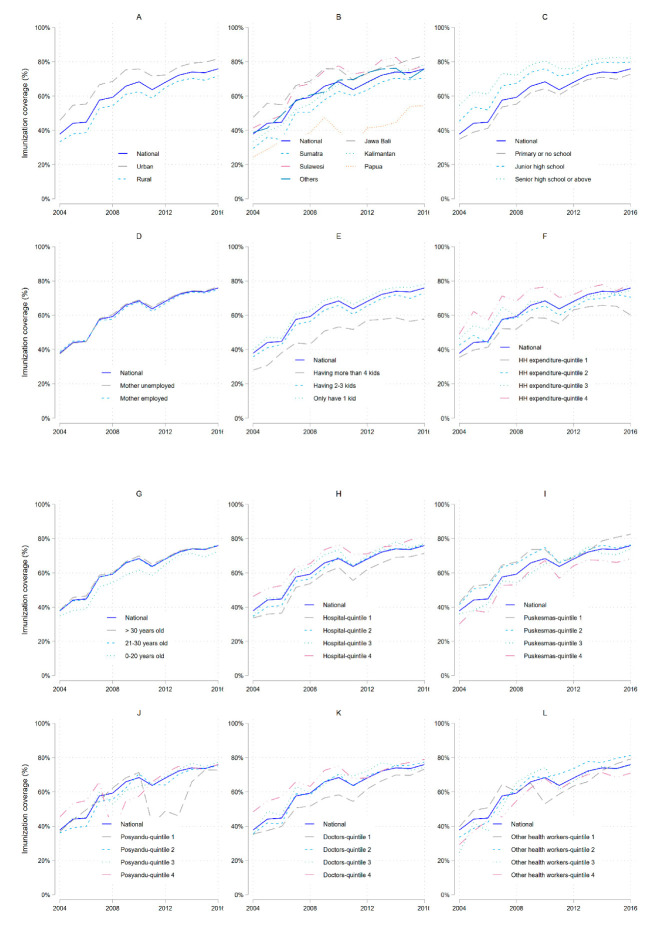
DTP3 immunization coverage by (**A**) residential area; (**B**) residential island; (**C**) mother’s education; (**D**) mother’s employment; (**E**) number of children; (**F**) household expenditure (in quintiles); (**G**) mother’s age; (**H**) density of hospitals (in quintiles); (**I**) density of puskesmas (in quintiles); (**J**) density of posyandu (in quintiles); (**K**) density of doctors (in quintiles); (**L**) density of other health workers (in quintiles).

The sociodemographic and district factors from the data on 305,090 children between 12 and 23 months of age from 2004 to 2016 are indicated in Figure 1 and Table S1. The proportion of children completely immunized with DTP3 increased from 37.8% in 2004 to 75.9% in 2016. However, the complete immunization rate differed on different islands. The rate of children born under the care of a professional birth attendant increased from 70.92% in 2004 to 90.01% in 2016. In 2004, the majority of mothers were 21–30 years old and had no education or only a primary school education. However, the proportion of mothers in that educational group decreased from 70.52% in 2004 to 50.55% in 2016. Families with 1–2 children were the most common, and this remained largely constant between 2004 and 2016. The percentage of employed mothers increased from 28.90% in 2004 to 42.89% in 2016. The mean household income also increased. With regard to medical resources, the numbers of hospitals, health posts, and health workers per 1000 population increased every year. The numbers of health centers and doctors per 1000 population also increased slightly.

The coverage of DTP3 immunization from 2004 to 2016 was always higher in urban areas, among mothers with a high school education or above, mothers with only one child, the families with household expenditure (HH expenditure) in the fourth quintile, and mothers >30 years old. DTP3 immunization coverage on the islands of Papua and Sumatra were consistently below the national coverage during that entire period. However, little difference was shown between mothers who were and were not employed.

At the district level, households with expenditures in the first quintile receiving care from puskesmas had higher coverage than those in other categories. Households with expenditures in the fourth quintile receiving care from doctors had the highest DTP3 immunization coverage.

**Figure 2:**
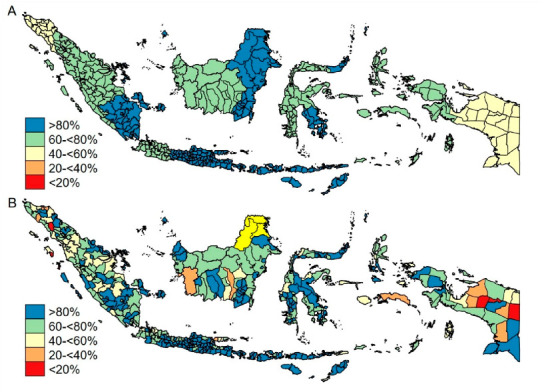
Spatial distribution of DTP3 immunization coverage by (**A**) provinces and (**B**) districts (2016).

Figure 2 shows that more than 80% of children in 249 districts received DTP3 immunization in 2016, especially children in the provinces South Sumatra, East and South Kalimantan, almost provinces in Java and Bali Island and North and Southeast Sulawesi.

**Figure 3:**
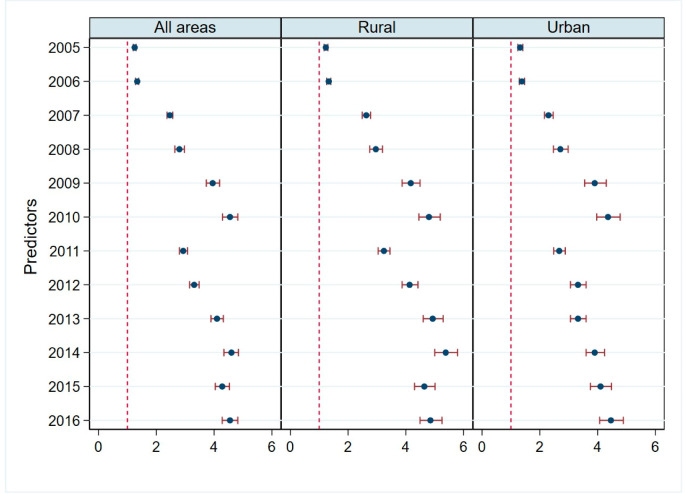
Children’s DTP immunization status in rural and urban areas, 2004–2016. (Notes: Results from multilevel models with 2004 as the reference. Controlled for island of residences, birth attended by a health professional, mother’s age, mother’s educational level, mothers employment status, number of children, household income, and district levels (hospitals/1000 population, health centers/1000 population, integrated health services/1000 population, doctors/1000 population, other health workers/1000 population). Reported are odds ratios (95% confidence intervals)).

We compared the complete DTP3 immunization rates between 2004 and each other year according to rural or urban residential status (Figure 3; Figure S2). This comparison indicated that the complete DTP3 immunization rate increased year by year and that the trend of increase was the same in rural and urban areas.

Table 1 indicates the relationships between the determinants and complete DTP3 immunization rates in the years 2004, 2010, and 2016 using multilevel logistic regression analysis. Residential area, support by a health professional during labor, mother’s age, mother’s education, mother’s working status, number of children, household income, and density of medical resources were the factors that significantly affected complete DTP3 immunization rates. Of all of these, the most effective factor in children’s DTP3 immunization rate was the presence of a professional birth attendant during labor.

We analyzed the relationship between island of residence and complete DTP3 immunization rate. With the exception of 2010, the complete immunization rate was the same among the islands. In 2010, Sulawesi Island had the highest rate.

We also evaluated the relationship between health care facility density and DTP3 immunization status by island of residence (Table 2). The factors relevant to the complete DTP3 immunization rates differed according to island of residence. In Papua, complete DTP3 immunization rates increased with increased health post density. However, increased densities of doctors and other health workers caused complete DTP3 immunization rates to decline. In Java, increased densities of health centers and of health workers other than doctors diminished the complete DTP3 immunization rate. In Sumatera, there was a positive relationship between the densities of health posts and doctors and complete DTP3 immunization rate. In Kalimantan, there was a negative relationship between densities of hospitals and health centers and complete DTP3 immunization rate, and a positive relationship between density of other health workers and immunization rate. In Sulawesi, there was a negative relationship between the densities of health centers and other health workers and complete DTP3 immunization rate.

Compared with other immunizations, in 2004, the proportion of children receiving BCG immunization (86%) was highest, followed by measles (79%). The proportions of children receiving DPT and polio immunization were similar (37% and 38%, respectively). Only approximately one quarter of children received hepatitis B immunization in the same year. The proportions of children receiving immunization increased significantly over 12 years. The increase from 2004 to 2016 was greatest for hepatitis B immunization (almost threefold), while the proportions of children receiving DPT and polio doubled during the same period (Table 3).

## 4. Discussion

The rate of complete DTP3 immunization in Indonesia has greatly increased every year, from 37.8% in 2004 to 75.9% in 2016 (Figure 1; Table S1). We evaluated the factors affecting improvement in the immunization rate in Indonesia, focusing on socioeconomic factors and density of medical resources. We concluded that complete immunization rates were significantly higher among children born with a professional birth attendant present than among those born without. Children whose births were supported by a professional birth attendant were reported as having a higher probability of receiving complete DTP3 immunization both in rural and urban areas [15,16,17]. One of the plausible explanations for this result is that professional birth attendants give parents appropriate information about maternal and newborn health, including information on immunization. In Indonesia, professional birth attendants not only assist in the delivery process but also provide antenatal care, postnatal care, nutrition advice, reproductive advice, and immunization services [17,18,19]. Furthermore, postnatal care by a professional with the same cultural background (from the same island) has been found particularly helpful to establishing well-baby care and receiving immunizations [20,21,22].

The children of older mothers were more likely to receive complete DTP3 immunization. Previous research has indicated that women in Indonesia under the age of 20 are less likely to immunize their children than older women [6]. Another analysis reported that children born to mothers younger than 20 were less likely to be vaccinated than the children of mothers older than 20 [23]. Teenage motherhood tends to increase the risk for poor maternal and infant outcomes, and younger women have more difficulty making decisions about their children’s health. Furthermore, having an older husband leads to fewer decisions being made by women [23].

Maternal educational level is important for children’s complete DTP3 immunization rates. This trend also concerns almost all types of immunization [22,24,25,26]. A mother’s education influences health-seeking behaviors, which include child immunization [24]. Mothers with more formal education have been shown to be more receptive to health information and modern ideas and to be more confident raising their children and making decisions regarding their families’ health [16,18,27,28]. Additionally, information related to preventive health is more readily accepted by mothers with higher levels of education [29,30].

Employed mothers were less likely to immunize their children than mothers who did not work outside the home. Employed mothers have been shown to have less time for and interest in their children than those who do not engage in paid work [16]. Previous studies suggest that employed mothers have less time for childcare than stay-at-home mothers [31]. On the other hand, employed mothers have more stressors and fatigue due to their employment and their household chores [31]. Mothers who spend longer hours at the workplace have less energy to devote to their children’s health needs, including following immunization schedules.

There are widening gaps in DTP3 immunization coverage between small and large families. Previous research has shown that larger families have a lower probability of obtaining complete immunization for their children [6,32,33]. A mother with many children is busy attending to her family’s needs, and her attention is divided among the children [16]. Additionally, mothers with many children are busy fulfilling their needs and become careless about vaccination schedules [24]. Our results indicate that having many children interferes with obtaining complete DTP3 immunization.

High household income was positively correlated with complete DTP3 immunization in this study. Previous research from many regions indicated that children from wealthier families are more likely to receive immunization than children from low-income families [21,26,34,35]. In Indonesia, although the government offers free DTP3 immunization services, some effort and financial resources are required to reach a health facility that provides DTP3 immunization.

This financial cost could be a barrier to immunization [26]. Furthermore, wealth index is correlated with better health status and health-seeking behaviors.

On the other hand, the density of medical resources was not always associated with a higher rate of complete DTP3 immunization in this study. The density of hospitals and integrated health posts was significantly associated with higher immunization rates. However, number of health centers and doctors were irrelevant to immunization rates, and higher numbers of health workers were correlated to lower immunization rates. Some people who come from wealthy families believe that they will receive a better-quality vaccine at a hospital than at a health post. Additionally, some people from wealthy families believe that the imported vaccines offered at hospitals are less likely to produce side effects [6]. Therefore, increasing the number of hospitals could promote DTP3 immunization rates.

In all areas of Indonesia, the density of integrated health posts influences the likelihood that children will receive complete DTP3 immunization. Health posts are managed by integrated health service post volunteers, or cadres. Cadres are selected from among the community members to organize integrated health service post activities. They know their communities well. Cadres therefore assist village midwives in distributing information, and they encourage and invite mothers to join the immunization program [36,37,38].

Increasing the number of health centers reveals no significant relationship with complete DTP3 immunization rates. Previous research indicated that increasing the number of health centers was not an effective way to increase immunization coverage [6]. Increasing the number of health centers could, however, be effective if the numbers of physicians and other health workers also increased along with sufficient equipment for carrying out immunization programs [39]. The quality and distribution of health centers could therefore be related to immunization. If access to facilities is not convenient and staffing is insufficient, health centers cannot contribute immunization regardless of whether the number of health centers increases. Health centers must provide healthcare services easily, and they should ideally be located within walking distance.

Additionally, even the number of doctors did not affect the probability of children receiving complete DTP3 immunization. The presence of doctors is the primary key to achieving health development and improving immunization coverage [40]. Indonesia is facing the problem of maldistribution of doctors. Thirty of Indonesia’s 33 provinces have not reached the number of doctors per 1000 population recommended by the WHO. Health facilities in rural areas are not considered ideal places for doctors to gain professional experience [41]. Doctors are therefore concentrated in urban areas, especially on the island of Java [42]. Only 20% of doctors are practicing in rural areas [42]. The imbalanced distribution of doctors may be one reason for which immunization rates are not affected by the number of doctors.

In general, health workers are the primary care providers in health centers, and their presence is an essential component in increasing DTP3 immunization coverage. However, in the present study, there was a negative relationship between density of health workers and complete DTP3 immunization rate. According to data from the Indonesian Ministry of Health, the majority of health workers are located on Java and Sumatra [43]. According to a report from the World Bank, there are shortages of both nurses and midwives in Indonesia: shortages of 87,874 and 15,311, respectively, in hospitals, and of 10,146 and 4485, respectively, in health centers [42]. Therefore, although the number of health workers increases, there is a shortage of health workers in both hospitals and health centers.

### Study Strengths and Limitations

This study has several strengths. Firstly, we used several large Indonesian data sources that included a representative sample from a population-based survey and covered every district in Indonesia. Secondly, we used multiyear data, which can capture changes each year. Thirdly, we executed multilevel analysis capable of capturing household-level and district-level factors and analyzed the data according to urban and rural conditions. This study was therefore able to capture real conditions concerning immunization in Indonesia. We also detected that unique geographical conditions and cultures could affect DTP3 immunization coverage.

On the other hand, there are some limitations to our study. Indonesia is the largest archipelago in the world and is home to the fourth-largest population in the world. The economic conditions and geographical features also vary among the islands [43]. We indicated that the determinants relevant to DTP3 immunization rates differed according to island of residence (see Table 2). However, we are unable to indicate the reasons for these differences. In addition, we believed that culture and characteristics could be related to the differences in immunization rate. However, since we could not indicate the reasons for which some determinants are influential, we intend in the future to collect data and material related to immunization on each island. This study used a cross-sectional design, which makes it difficult to point to the directionality of causal relationships between immunization and its factors. Lastly, the immunization data we used was based on parent interviews. In general, parents were not asked to show their children’s immunization cards. Since 2015, they have been asked to show the immunization cards at the interviews, but when parents do not have immunization cards, they are permitted to answer according to their memory. Therefore, our data could include recall biases due to the fallibility of memory.

## 5. Conclusions

Both household-level and district-level factors are essential in increasing DTP3 immunization coverage in Indonesia. Generally, awareness regarding DTP3 immunization is higher among families whose children’s births were aided by professional birth attendants, among small families, and among wealthy families. At the district level, the factors related to DTP3 immunization coverage differ between rural and urban areas and among islands.

As such, district governments must identify local problems and create innovative programs to increase DTP3 immunization coverage. Furthermore, the central government should strongly advocate supporting local stakeholders in their efforts to ensure efficient and effective management of immunization programs.

## Figures and Tables

**Table 1:** Determinants of children’s DTP immunization status.

	2004	2010	2016	All Years
Island of residence Papua	Reference	Reference	Reference	Reference
Java-Bali	1.2 (0.61–2.36)	4.38 (2.25–8.51) **	1.53 (0.94–2.48)	4.46 (3.34–5.97) **
Sumatra	0.79 (0.43–1.44)	2.03 (1.1–3.73) *	0.81 (0.52–1.25)	2.14 (1.62–2.83) **
Kalimantan	1.00 (0.54–1.87)	2.52 (1.32–4.79) *	1.47 (0.93–2.33)	2.59 (1.89–3.55) **
Sulawesi	1.85 (0.99–3.44)	5.65 (2.99–10.7) **	1.53 (0.97–2.41)	4.37 (3.24–5.9) **
Other islands (Nusa Tenggara, Maluku)	1.17 (0.6–2.28)	4.23 (2.2–8.13) **	1.25 (0.78–1.99)	3.1 (2.26–4.27) **
Living area Rural	Reference	Reference	Reference	Reference
Urban	1.14 (1.05–1.23) *	1.19 (1.1–1.28) **	1.24 (1.11–1.37) **	1.16 (1.13–1.18) **
Birth attended by health professional No	Reference	Reference	Reference	Reference
Yes	1.57 (1.46–1.7) **	1.61 (1.48–1.75) **	1.97 (1.71–2.26) **	1.53 (1.5–1.56) **
Mother’s age 20 years	Reference	Reference	Reference	Reference
21–30 years	1.15 (1.02–1.29) *	1.19 (1.05–1.35) *	1.3 (1.09–1.54) *	1.15 (1.11–1.19) **
>30 years	1.13 (0.99–1.29)	1.32 (1.15–1.53) **	1.44 (1.2–1.73) **	1.27 (1.22–1.32) **
Mother’s educational level Primary	Reference	Reference	Reference	Reference
Secondary	1.21 (1.13–1.31) **	1.34 (1.24–1.46) **	1.3 (1.18–1.44) **	1.3 (1.27–1.33) **
Higher	1.37 (1.19–1.58) **	1.43 (1.26–1.63) **	1.42 (1.24–1.63) **	1.39 (1.34–1.44) **
Mother’s working status No	Reference	Reference	Reference	Reference
Yes	1.07 (1.00–1.15) *	1.12 (1.04–1.2) *	0.99 (0.9–1.08)	1.04 (1.02–1.06) **
Number of children Few	Reference	Reference	Reference	Reference
Intermediate	0.92 (0.85–0.99) *	0.89 (0.82–0.97) *	0.85 (0.77–0.95) *	0.87 (0.86–0.89) **
Many	0.72 (0.61–0.84) **	0.62 (0.52–0.73) **	0.56 (0.44–0.72) **	0.66 (0.64–0.7) **
Household income	1.38 (1.29–1.48) **	1.36 (1.26–1.46) **	1.11 (1.03–1.21) *	1.27 (1.24–1.29) **
Medical resources/1000 population				
Hospitals	1.19 (1.05–1.34) *	1.2 (1.04–1.39) *	1.19 (1.06–1.33) *	0.95 (0.47–1.91)
Health centers	0.41 (0.16–1.09)	0.88 (0.36–2.15)	0.63 (0.34–1.16)	0.94 (0.79–1.11)
Village health posts	2.36 (1.76–3.16) **	0.91 (0.59–1.41)	1.37 (1.12–1.69) *	1.19 (1.15–1.23) *
Doctors	1.58 (0.8–3.13)	0.84 (0.44–1.62)	0.66 (0.35–1.26)	1.2 (1.07–1.35) *
Other health workers	0.65 (0.45–0.93) *	1.03 (0.9–1.18)	0.91 (0.84–0.99) *	0.97 (0.95–0.98) *
Between-district variance	0.17	0.24	0.53	0.16
ICC	0.67	1.06	0.13	0.61
Median odds ratio	2.19	2.67	2.	2.11

Note: Reported are odds ratios (95% confidence intervals). * *p* < 0.05; ** *p* < 0.001.

**Table 2:** Determinants of children’s DTP immunization status by island (Model 2).

Island	Hospitals/1000 pop	Health Centers/1000 pop	Integrated Health Posts/1000 pop	Doctors/1000 pop	Other Health Worker/1000 pop
Papua	2.52 (0.21–29.73)	1.82 (1.26–1.52) *	1.61 (1.37–1.9) **	0.64 (0.46–0.89) *	0.86 (0.81–0.91) **
Java	4.13 (0.91–18.82)	3.02 (1.09–8.41) *	1.06 (0.97–1.16)	0.93 (0.71–1.2)	1.19 (1.08–1.31) **
Sumatera	1.65 (0.59–4.63)	0.9 (0.64–1.25)	1.39 (1.3–1.49) **	1.24 (1.03–1.51) *	0.99 (0.96–1.03)
Kalimantan	0.07 (0.00–0.84) *	0.52 (0.34–0.81) *	1.01 (0.91–1.13)	1.26 (0.82–1.94)	1.09 (1.04–1.16) *
Sulawesi	5.75 (0.37–89.55)	0.52 (0.36–0.76) *	1.07 (0.97–1.18)	1.22 (0.84–1.78)	0.95 (0.91–0.99) *

Note: Controlled for rural/urban living area, birth attended by health professional, mother’s age, mother’s educational level, mother’s working status, number of children, and household income. Reported are odds ratios (95% confidence intervals). Sig: * *p* < 0.05, ** *p* < 0.001.

**Table 3:** Immunization status for each immunization type in 2004 and 2016.

	2004 *n* (%)	2016 *n* (%)
DTP		
Complete	10,231 (37.83)	14,329 (75.93)
Non complete	16,814 (62.17)	4542 (24.07)
Polio		
Complete	10,338 (38.23)	14,924 (79.24)
Non complete	16,703 (61.77)	3911 (20.76)
Hepatitis B		
Complete	6948 (25.70)	13,649 (75.37)
Non complete	20,085 (74.30)	4461 (24.63)
BCG		
Complete	23,444 (86.70)	17,472 (92.89)
Non complete	3596 (13.30)	1337 (7.11)
Measles		
Complete	21,371 (79.05)	16,003 (85.56)
Non complete	5664 (20.95)	2701 (14.44)

## Supplementary Materials

The following are available online at https://www.mdpi.com/2076-393X/8/3/518/s1, Figure S1: Children’s DTP3 immunization coverage by year, Table S1: Descriptive statistics on household and district characteristics.
