# Supplementary material for: Trends, Spatial Disparities, and Social Determinants of DTP3 Immunization Status in Indonesia 2004–2016
Source: Vaccines (Basel). 2020 Sep 10;8(3):518. doi: 10.3390/vaccines8030518 (PMC7563731; doi:10.3390/vaccines8030518)
Supplement: Supplementary file 1 [file vaccines-08-00518-s001.zip › suplementary/Table S1Descriptive statistics on household and district characteristics.docx]

**Table 1, Descriptive statistics on household and district characteristics**

|  | 2004 | 2005 | 2006 | 2007 | 2008 | 2009 | 2010 | 2011 | 2012 | 2013 | 2014 | 2015 | 2016 |
| --- | --- | --- | --- | --- | --- | --- | --- | --- | --- | --- | --- | --- | --- |
| Children receiving complete DTP immunization (n(%)) | 10,231 (37.83) | 10,171 (44.12) | 10,315 (44.77) | 15,109 (57.60) | 15,557 (59.27) | 17,585 (65.79) | 18,984 (68.34) | 15,135 (63.71) | 15,180 (68.07) | 14,855 (72.14) | 15,179 (74.02) | 13,941 (73.63) | 14,329 (75.93) |
| **Residential island** (n(%)) | | | | | | | | | | | | | |
| Papua | 812 (3.00) | 451 (1.96) | 769 (3.34) | 853 (3.25) | 922 (3.51) | 1,014 (3.79) | 1,260 (4.54) | 1,798 (7.57) | 1,396 (6.26) | 1,285 (6.24) | 1,240 (6.05) | 919 (4.85) | 1,031 (5.46) |
| Java and Bali | 7,119 (26.32) | 6,359 (27.58) | 5,753 (24.97) | 6,644 (25.33) | 6,699 (25.52) | 6,876 (25.72) | 6,769 (24.37) | 6,210 (26.14) | 6,278 (28.15) | 5,657 (27.47) | 5,669 (27.64) | 5,394 (28.49) | 5,458 (28.92) |
| Sumatra | 7,514 (27.78) | 6,390 (27.72) | 7,201 (31.25) | 7,994 (30.47) | 8,152 (31.06) | 8,260 (30.90) | 9,097 (32.75) | 7,030 (29.59) | 6,527 (29.27) | 5,968 (28.98) | 5,975 (29.13) | 5,631 (29.74) | 5,442 (28.83) |
| Kalimantan | 3,029 (11.20) | 2,803 (12.16) | 2,448 (10.62) | 2,911 (11.10) | 3,010 (11.47) | 3,004 (11.24) | 3,010 (10.84) | 2,458 (10.35) | 2,183 (9.79) | 1,989 (9.66) | 2,019 (9.84) | 1,876 (9.91) | 1,844 (9.77) |
| Sulawesi | 3,879 (14.34) | 4,100 (17.79) | 3,900 (16.93) | 4,472 (17.05) | 4,204 (16.02) | 4,158 (15.55) | 4,139 (14.90) | 3,183 (13.40) | 3,059 (13.72) | 2,954 (14.35) | 2,959 (14.43) | 2,788 (14.73) | 2,769 (14.67) |
| Other islands | 4,692 (17.35) | 2,950 (12.80) | 2,970 (12.89) | 3,358 (12.80) | 3,262 (12.43) | 3,419 (12.79) | 3,504 (12.61) | 3,077 (12.95) | 2,858 (12.82) | 2,738 (13.30) | 2,646 (12.90) | 2,325 (12.28) | 2,329 (12.34) |
| **Living in urban area** (n(%)) | 9,710 (35.90) | 8,511 (36.92) | 8,452 (36.68) | 9,030 (34.42) | 9,026 (34.39) | 8,934 (33.42) | 12,038 (43.33) | 9,137 (38.46) | 9,240 (41.43) | 8,361 (40.61) | 8,395 (40.94) | 7,922 (41.84) | 7,954 (42.15) |
| **Birth attended by health professional** (n(%)) | 19,173 (70.92) | 15,857 (68.79) | 16,050 (69.66) | 18,174 (69.28) | 18,641 (71.06) | 19,544 (73.11) | 21,413 (77.08) | 17,981 (75.69) | 17,714 (79.43) | 16,924 (82.19) | 17,322 (84.47) | 14,662 (88.18) | 15,094 (90.01) |
| **Mother’s age** (n(%)) | | | | | | | | | | | | | |
| 20 years | 2,394 (8.85) | 1,469 (6.37) | 1,491 (6.47) | 1,798 (6.85) | 1,683 (6.41) | 1,715 (6.42) | 1,845 (6.64) | 1,468 (6.18) | 1,297 (5.82) | 1,273 (6.18) | 1,242 (6.06) | 1,172 (6.19) | 1,043 (5.53) |
| 21-30 years | 14,103 (52.15) | 12,527 (54.34) | 12,549 (54.46) | 14,204 (54.15) | 14,007 (53.36) | 14,280 (53.42) | 15,015 (54.05) | 12,518 (52.69) | 11,354 (50.91) | 10,010 (48.61) | 9,814 (47.85) | 8,931 (47.17) | 8,888 (47.10) |
| >30 years | 10,548 (39.00) | 9,057 (39.29) | 9,001 (39.07) | 10,230 (39.00) | 10,559 (40.23) | 10,736 (40.16) | 10,919 (39.31) | 9,770 (41.13) | 9,650 (43.27) | 9,308 (45.20) | 9,452 (46.09) | 8,830 (46.64) | 8,940 (47.37) |
| **Mother’s education** (n(%)) | | | | | | | | | | | | | |
| Primary/no education | 17,950 (70.52) | 15,889 (68.92) | 14,853 (66.82) | 16,806 (66.18) | 16,039 (64.04) | 16,329 (63.10) | 15,686 (58.50) | 13,784 (60.56) | 12,534 (57.96) | 11,366 (56.91) | 11,015 (55.25) | 10,109 (54.79) | 9,372 (50.55) |
| Secondary | 6,231 (24.48) | 5,683 (24.65) | 5,825 (26.21) | 6,457 (25.43) | 7,029 (28.07) | 6,949 (26.86) | 7,910 (29.50) | 6,384 (28.05) | 6,345 (29.34) | 5,945 (29.77) | 5,936 (29.77) | 5,763 (31.24) | 5,957 (32.13) |
| Higher | 1,271 (4.99) | 1,481 (6.42) | 1,550 (6.97) | 2,130 (8.39) | 2,337 (9.33) | 2,598 (10.04) | 3,218 (12.00) | 2,592 (11.39) | 2,747 (12.70) | 2,662 (13.33) | 2,986 (14.98) | 2,577 (13.97) | 3,211 (17.32) |
| **Employed mothers** (n(%)) | 7,750 (28.90) | 6,987 (30.31) | 6,678 (28.98) | 10,548 (40.21) | 11,084 (42.23) | 10,737 (40.17) | 11,570 (41.65) | 10,216 (43.01) | 9,384 (42.08) | 8,752 (42.50) | 8,925 (43.52) | 8,123 (42.91) | 8,094 (42.89) |
| **Number of children** (n(%)) | | | | | | | | | | | | | |
| 1-2 children | 15,696 (60.12) | 13,731 (59.56) | 13,706 (59.49) | 15,432 (58.83) | 15,739 (59.96) | 16,250 (60.79) | 17,452 (62.82) | 14,834 (62.46) | 13,911 (62.38) | 12,847 (62.39) | 12,700 (61.93) | 12,517 (66.11) | 12,438 (65.91) |
| 3-5 children | 8,786 (33.66) | 8,043 (34.89) | 7,893 (34.26) | 9,099 (34.69) | 8,888 (33.86) | 8,903 (33.31) | 8,906 (32.06) | 7,603 (32.02) | 7,206 (32.31) | 6,711 (32.59) | 6,793 (33.13) | 5,821 (30.75) | 5,897 (31.25) |
| >5 children | 1,624 (6.22) | 1,279 (5.55) | 1,442 (6.26) | 1,701 (6.48) | 1,622 (6.18) | 1,578 (5.90) | 1,421 (5.12) | 1,311 (5.52) | 1,183 (5.30) | 1,033 (5.02) | 1,013 (4.94) | 595 (3.14) | 536 (2.84) |
| **Household income**  **(x1000 IDR) (Mean(SD))** | 830.7 (810.60) | 1,037 (1,067.9) | 1,201.4 (931) | 1,329.8 (862.9) | 1,894 (1,669.7) | 1,827.7 (1,502) | 2,147.9 (1,776.3) | 2,401.4 (2,456.0) | 2,751.6 (3,502.8) | 2,935.6 (3,547.4) | 3,234.6 (3,543.1) | 3,174.9 (3,110.2) | 3,867.5 (3382.2) |
| **Medical resources/1000 pop (District level) (Mean(SD))** | | | | | | |  |  |  |  |  |  |  |
| Hospitals |  | 0.03 (0.03) |  |  | 0.02 (0.02) |  |  | 0.03 (0.03) |  |  | 0.02 (0.02) |  |  |
| Health centers (*Puskesmas*) |  | 0.23 (0.16) |  |  | 0.24 (0.2) |  |  | 0.23 (0.20) |  |  | 0.22 (0.21) |  |  |
| Integrated health service posts (*Posyandu*) |  | 1.05 (0.37) |  |  | 0.49 (0.41) |  |  | 1.39 (0.60) |  |  | 1.20 (0.51) |  |  |
| Doctors |  | 0.19 (0.17) |  |  | 0.26 (0.21) |  |  | 0.20 (0.19) |  |  | 0.21 (0.16) |  |  |
| Other health workers |  | 0.84 (0.48) |  |  | 2.51 (1.4) |  |  | 1.60 (1.00) |  |  | 1.96 (1.4) |  |  |
