# Supplementary figures and images for: Trends, Spatial Disparities, and Social Determinants of DTP3 Immunization Status in Indonesia 2004–2016
Source: Vaccines (Basel). 2020 Sep 10;8(3):518. doi: 10.3390/vaccines8030518 (PMC7563731; doi:10.3390/vaccines8030518)

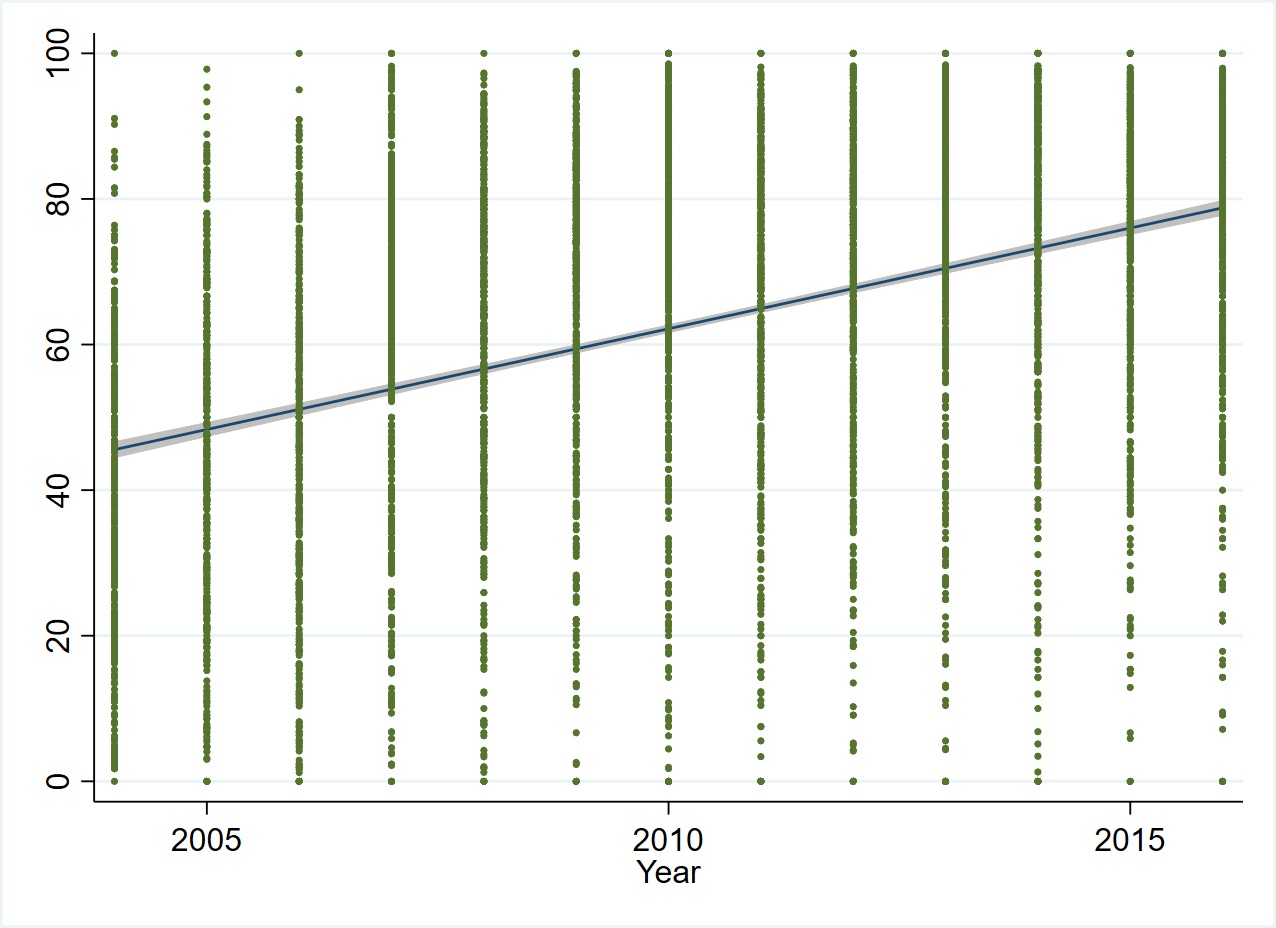

Supplement: Supplementary file 1 [file vaccines-08-00518-s001.zip › suplementary/FigureS1Childrens DTP3 immunization coverage by year..jpeg]
